# Effects of eccentric, concentric and eccentric/concentric training on muscle function and mass, functional performance, cardiometabolic health, quality of life and molecular adaptations of skeletal muscle in COPD patients: a multicentre randomised trial

**DOI:** 10.1186/s12890-022-02061-4

**Published:** 2022-07-19

**Authors:** Luis Peñailillo, Denisse Valladares-Ide, Sebastián Jannas-Velas, Marcelo Flores-Opazo, Mauricio Jalón, Laura Mendoza, Ingrid Nuñez, Orlando Diaz-Patiño

**Affiliations:** 1 Exercise and Rehabilitation Sciences Laboratory, School of Physical Therapy, Faculty of Rehabilitation Sciences, Universidad Andres Bello, 700 Fernández Concha, Las Condes, 7591538 Santiago, Chile; 2 Long Active Life Laboratory, Instituto de Ciencias de la Salud, Universidad de O’Higgins, Rancagua, Chile; 3 Instituto Nacional del Tórax, Santiago, Chile; 4 Respiratory Unit, Departamento de Medicina, Hospital Clínico Universidad de Chile, Santiago, Chile; 5 Department of Pulmonary Diseases, Faculty of Medicine, Pontifical Catholic University of Chile, Santiago, Chile; 6 Department of Critical Care, Faculty of Medicine, Pontifical Catholic University of Chile, Santiago, Chile; 7 Institute of Health Sciences, Universidad de O´Higgins, Rancagua, Chile

**Keywords:** Chronic obstructive pulmonary disease, Aerobic training, Respiratory disease

## Abstract

**Background:**

Chronic obstructive pulmonary disease (COPD) is the third cause of death worldwide. COPD is characterised by dyspnoea, limited exercise tolerance, and muscle dysfunction. Muscle dysfunction has been linked to dysregulation between muscle protein synthesis, myogenesis and degradation mechanisms. Conventional concentric cycling has been shown to improve several clinical outcomes and reduce muscle wasting in COPD patients. Eccentric cycling is a less explored exercise modality that allows higher training workloads imposing lower cardio-metabolic demand during exercise, which has shown to induce greater muscle mass and strength gains after training. Interestingly, the combination of eccentric and concentric cycling training has scarcely been explored. The molecular adaptations of skeletal muscle after exercise interventions in COPD have shown equivocal results. The mechanisms of muscle wasting in COPD and whether it can be reversed by exercise training are unclear. Therefore, this study aims two-fold: (1) to compare the effects of 12 weeks of eccentric (ECC), concentric (CONC), and combined eccentric/concentric (ECC/CONC) cycling training on muscle mass and function, cardiometabolic health, physical activity levels and quality of life in severe COPD patients; and (2) to examine the molecular adaptations regulating muscle growth after training, and whether they occur similarly in specific muscle fibres (i.e., I, IIa and IIx).

**Methods:**

Study 1 will compare the effects of 12 weeks of CONC, ECC, versus ECC/CONC training on muscle mass and function, cardiometabolic health, levels of physical activity and quality of life of severe COPD patients using a multicentre randomised trial. Study 2 will investigate the effects of these training modalities on the molecular adaptations regulating muscle protein synthesis, myogenesis and muscle degradation in a subgroup of patients from Study 1. Changes in muscle fibres morphology, protein content, genes, and microRNA expression involved in skeletal muscle growth will be analysed in specific fibre-type pools.

**Discussion:**

We aim to demonstrate that a combination of eccentric and concentric exercise could maximise the improvements in clinical outcomes and may be ideal for COPD patients. We also expect to unravel the molecular mechanisms underpinning muscle mass regulation after training in severe COPD patients.

*Trial Registry*: Deutshches Register Klinischer Studien; Trial registration: DRKS00027331; Date of registration: 12 January 2022. https://www.drks.de/drks_web/navigate.do?navigationId=trial.HTML&TRIAL_ID=DRKS00027331.

## Background

Chronic obstructive pulmonary disease (COPD) is the third leading cause of death worldwide [1, 2]. Clinically, COPD is primarily characterised by airflow limitation resulting from inflammation and remodelling of the airways associated with parenchymal destruction emphysema [3]. Consequentially, COPD impairs functional capacity, decreases mobility, and promotes sedentary behaviour (i.e., disuse) due to the progressive worsening of dyspnea [4]. In fact, the inability to be physically active is one of the frequent complaints expressed by patients with COPD, which represents a downward spiral that aggravates the systemic consequences of the disease due to disuse [5]. Furthermore, patients with COPD frequently develop metabolic and cardiac complications (i.e., insulin resistance, dyslipidaemia, hypertension, etc.), which in association with the above-mentioned clinical manifestations, results in reduced quality of life [3] and an increased mortality rate in COPD patients in comparison to their healthy counterparts [6].

A common extra-pulmonary manifestation in patients with COPD is the lower limb muscle dysfunction**,** which includes structural and morphological alterations compromising muscle function and mass, as evidenced by reduced muscle endurance and strength [7]. The reduced muscle endurance capacity in COPD patients is the result of impaired muscle oxidative capacity, a shift toward a glycolytic muscle fibre type (type II) distribution (i.e., a low fraction of type I fibres) [8], reduced capillarity (i.e., low capillary to fibre ratio) and reduced cross-sectional area (CSA) of all muscle fibre types reported in COPD [9]. These changes are clinically manifested by reduced exercise endurance capacity and increased fatigability [3], which also limit daily life activities and exacerbate poor health status in COPD [10]. The reduced muscle strength is evidenced by an accelerated decline in lower limb muscle strength in COPD compared to healthy counterparts (4% vs. 1–2% per year) [11]. The muscle strength loss has been closely associated with an involuntary muscle mass loss or muscle wasting [11], which affects 10–40% of COPD patients [12] and has been related to multiple complications such as poorer survival [13–15], and increased hospital readmission, need for mechanical ventilator support (i.e., health care resources), morbidity, and mortality [3, 16]. Furthermore, muscle wasting and dysfunction are clinically associated with decreased functional capacity (i.e., 6-min walking test; 6MWT) [17, 18], increased risk of falls (i.e., timed up and go; TUG), limited mobility and daily physical activity, which promotes sedentary behaviour, and decrease quality of life in COPD patients [19].

### Exercise in pulmonary rehabilitation for COPD

Pulmonary rehabilitation (PR) has the challenge of reversing the deleterious impact of muscle wasting and dysfunction in conjunction with other systemic manifestations of COPD (e.g., hypertension, dyslipidaemia, and diabetes). However, exercise must accommodate the limitations of these patients. These limitations result from the inability to increase oxygen delivery to the peripheral muscles, gas exchange inefficiency in the lungs, constraints on lung mechanics (i.e., dynamic hyperinflation and flow limitation), and pulmonary hypertension during exercise [20]. Several clinical trials have shown that exercise markedly improves functional performance, health-related quality of life, perception of dyspnoea, muscle mass, and exercise capacity in COPD patients [21–23]. Endurance training has probably been the most common exercise modality prescribed for patients with COPD [24–26], which has shown to improve exercise tolerance, quality of life [26], muscle oxidative capacity [27], promote a fibre-type shift from type IIx to type I fibres [28], and increase CSA of all muscle fibre types [9]. Furthermore, it enhanced mitochondrial oxidative enzyme activity and increased capillary to muscle fibre ratio in patients with COPD [27]. However, there is also evidence that aerobic training may not be ideal for COPD as it could induce excessive oxidative stress and even decrease muscle mass in these patients [29–31]. On the other hand, resistance training has shown to increase muscle mass and strength in severe COPD patients, evidencing a preserved responsiveness to increases in muscle mass and strength in these patients [32]. Furthermore, resistance training has been shown to improve functional performance, mobility, and quality of life [33]. Although it is clear that exercise can reverse some of the clinical manifestations of COPD, the ventilatory constraints and lower limb muscle weakness of these patients limit their participation in traditional exercise, which reduces the extent of the benefits obtained from exercise. Thus, a modality of exercise to maximise the benefits for these patients is still a matter of study.

### Eccentric cycling training as a potentiator of PR in COPD

Eccentric cycling is a more novel and less explored training modality, in which eccentric training of the lower limb muscles is performed when resisting the backward rotational movement of the cranks generated by an eccentric ergometer. We have recently demonstrated that eccentric cycling is safe and could be ideal for COPD patients since it imposes lesser cardiopulmonary, metabolically, and perceptually demand (i.e., lower oxygen consumption, dyspnoea, and blood pressure) and allows greater workload production for the same metabolic demand than concentric cycling in COPD patients [34]. Moreover, when eccentric cycling is used as a training modality, it has shown to induce greater gains in muscle mass and strength [35, 36], and functional performance [37] in several clinical populations and COPD [35–37]. MacMillan et al. reported that 10-week eccentric cycling training increased muscle strength and mass in moderate COPD patients [35]. In line with that, Inostroza et al. [38] showed that 12 weeks of eccentric cycling training induced increases in muscle mass of the lower limbs and functional capacity, as shown by a 25% increase in the 6-min walking test, which was accompanied by improvements in quality of life of these patients. Thus, it seems that eccentric cycling training could be an ideal exercise modality for COPD. However, a limitation of eccentric cycling training is the modest adaptation to the oxidative metabolism after training [35, 39]. Interestingly, Steiner et al. showed that concentric cycling training increased mitochondrial volume density (14%) and increased aerobic capacity in COPD. However, muscle mass was decreased by 1%, suggesting that aerobic training may not be ideal for COPD [39]. Thus, as concentric cycling training has shown to induce metabolic improvements (i.e., insulin sensitivity, lipid profile, fat-mass, and mitochondrial adaptations), and eccentric cycling has shown to improve muscle mass and functional performance after training, it seems plausible to think that a combination of ECC and CONC (ECC + CONC) training could maximise the benefits of training for COPD patients.

### Muscle wasting in COPD

It is known that the Akt/mTOR/p70s6K/rS6 signalling pathway and the expression of myogenic regulatory factors (MRFs) (e.g., Myf5, Myod, myogenin) are essential mechanisms regulating muscle growth. It is known that the maintenance of skeletal muscle mass is dependent on the balance between the rates of muscle protein synthesis and degradation. However, this balance is tilted toward reduced protein synthesis and increased protein degradation, causing muscle wasting in COPD [40–42]. Muscle wasting has been associated with elevated systemic inflammation, oxidative stress, and physical deconditioning (e.g., disuse) of COPD patients [43, 44]. Intriguingly, some unclear results exist regarding the regulation of this balance, with studies reporting a decreased muscle protein synthesis rate in COPD patients [45], while others reported an increased protein synthesis in severe COPD when compared with healthy controls [46]. Additionally, MRFs and myostatin are also increased in COPD patients [47, 48]. This apparent contradictory regulation has been proposed to be an attempt of skeletal muscle to counterbalance an increased protein degradation in COPD patients.

### MicroRNAs as potential biomarkers of muscle dysfunction in COPD

In recent years, muscle-specific microRNAs (miRs) have been pointed out as crucial muscle growth regulators during disuse, overloading, and chronic diseases since they could play a significant role in proliferation and differentiation during myogenesis [10]. Farre-Garros et al. showed that muscle miR-542-3p and miR-542-5p were associated with muscle dysfunction and could suppress muscle protein synthesis in COPD [49]. Furthermore, Barreiro et al. showed that the levels of muscle miR-1, miR-206, miR-486, miR-29b, miR-133a, miR-27a, and miR-181a were lower in COPD patients with muscle wasting [50]. Interestingly, miR-208b and miR-499 have shown to be encoded in type I fibres only [51]. Thus, it seems that microRNAs are involved in muscle mass regulation, and their expression in muscle might be fibre-type specific [52]. Similarly, plasma levels of several muscle-specific miR (miR-1, miR-499, miR-181, miR133, and miR-206) have shown to be higher in patients with severe COPD than in controls [53, 54]. Furthermore, plasma miRs (miR-21, miR-146a, miR-221, miR-222) could be associated with exercise training [54, 55]. Thus, it appears that miRs in plasma could be an excellent biomarker to monitor muscle mass function and the effects of exercise in COPD patients. However, this has not been explored.

### Effects of exercise on skeletal muscle growth and degradation in COPD

Although muscle mass and strength have shown to increase after training in patients with severe COPD [32], this increase seems to be smaller than in control individuals. This may suggest a disconnection between changes in protein expression and muscle growth in COPD patients. However, acute aerobic exercise has shown to increase muscle protein degradation in COPD [26, 56, 57]. For instance, endurance exercise training (i.e., concentric cycling) increased the ubiquitin–proteasome (UP) system after training in COPD patients [58]. Also, Vogiatzis et al. reported greater ubiquitination after 12-week endurance training, which was associated with reduced protein synthesis in severe COPD patients [26]. Barreiro et al. also reported greater oxidative stress damage to muscle proteins after endurance training in severe COPD patients [29]. Therefore, it seems that endurance training could induce protein degradation in severe patients with COPD [26, 31]. The role of exercise on muscle autophagy has not been much investigated in COPD, but its activation depends on exercise intensity, volume, and modality [59–62]. The activation of autophagy with exercise may serve to enhance the removal of damaged proteins and organelles such as mitochondria [63, 64]. Additional research is needed to establish if improved autophagy can impact muscle oxidative metabolism and protein synthesis in COPD patients. Interestingly, Valladares-Ide et al. have recently shown that protein synthesis (mTOR and p70s6K phosphorylation) and MRF expression (MyoD and MRF4) increased, and markers for protein degradation (Atrogin-1 and MuRF-1) decreased after repeated bouts of eccentric cycling in young, healthy men [65]. They concluded that repeated eccentric cycling seems to induce a muscle-signalling response leading to muscle growth with limited muscle degradation signalling. Recently, eccentric exercise showed to stimulate muscle protein synthesis and decrease autophagy in an cachectic murine model, while no changes were observed after concentric exercise [66]. Thus, it is possible that eccentric exercise training could stimulate muscle protein synthesis to a greater extent than classic interventions such as concentric endurance training interventions in COPD.

### Muscle fibre-specific adaptations

It is known that human skeletal muscle contains a mixture of fibres exhibiting different contractile and metabolic properties. In general, the slow-twitch type I fibres possess a greater capacity for oxidative energy production, whereas the fast-twitch type II fibres demonstrate a larger glycolytic and force production capacity. Although the percentage of type I fibre is decreased and type II is increased in COPD, the fibre CSA is reduced more in type II fibres of COPD patients in comparison with healthy controls [52]. It has been shown that the fibre type and morphology can be changed depending on the exercise intervention performed [23]. There is growing evidence that muscle adaptations are encoded to a specific type of muscle fibres, and it seems that molecular adaptations to exercise are fibre type-specific, which depend upon the type and intensity of the exercise, and the involvement of type I and type II fibres in such exercise. This could explain equivocal results in COPD when analysing the synthesis and degradation pathways in whole muscle. Thus, it would be interesting to investigate whether exercise training regulates the molecular pathways for muscle growth and degradation in a fibre-specific manner in COPD.

Therefore, eccentric cycling is lesser cardiorespiratory and metabolic demanding than conventional endurance concentric cycling, which allows training at much higher workloads. Furthermore, eccentric cycling training in COPD patients improves functional capacity, muscle strength and mass. On the other hand, concentric training has shown improvements in the oxidative and metabolic capacity of COPD patients. Hence, there is a possibility that when combining eccentric and concentric cycling training, the adaptations in COPD could be maximised. Furthermore, there is a lack of knowledge on changes within skeletal muscles underlying these beneficial effects induced by exercise in COPD.

## Methods

### Aim

The aim of this project is two folds. To investigate the effects of eccentric and concentric, eccentric, and concentric cycling training on (1) muscle mass, muscle function, functional performance, blood markers of cardiometabolic health, physical activity levels and quality of life in patients with severe COPD; and (2) the regulatory pathways for muscle synthesis (Akt/mTOR/p70s6K/rS6), MFR expression, muscle degradation (UP and autophagy), and miRs expression in the whole muscle and a fibre-type specific manner after training.

### Experimental design

This project will investigate the effects of combined concentric and eccentric cycling training (ECC + CONC), purely eccentric (ECC), and purely concentric cycling (CONC) training on muscle mass, muscle function, functional performance, daily physical activity level and quality of life of severe COPD patients using a multicentre randomised clinical trial design. Furthermore, the study will compare the effects of exercise training on the regulatory pathways of muscle synthesis (Akt/mTOR/p70s6K/rS6), MFRs expression and muscle degradation (UP and autophagy) in a fibre-specific manner in severe COPD patients. We will examine the differential response to exercise training in whole muscle homogenates and isolated muscle fibres (type I and II muscle fibres) from VL muscle samples taken from a subgroup of patients from each training group (ECC + CONC, ECC, and CONC). Finally, we will assess the relationship between plasma and muscle miRs expression to investigate the validity of plasma miRs as biomarkers of muscle dysfunction.

### Experimental approach

We will compare the effects of CONC, ECC and the combination of CONC + ECC training on the changes in exercise performance (aerobic capacity and muscle strength), muscle mass, functional performance, levels of physical activity, quality of life, changes in biomarkers of cardiometabolic health (insulin resistance and lipid profile) and systemic inflammation. The proposed study is a multicentre randomised trial that will employ a 3 (groups) × 2 (measure times: Pre- and Post-) repeated measures design. The total duration of the proposed study is 14 weeks, including a 12-week training period and a testing week before and after the intervention. A stratified randomised assignment upon sequential number (by block) process will ensure that the experimental groups are balanced for FEV_1_, sex, and age. The allocation sequence will be performed by the multicentre coordinator. The training sessions will be conducted at three clinical centres in Santiago, Chile (Instituto Nacional del Torax, Hospital Clinico Universidad de Chile, Hospital Clinico Universidad Catolica). Assessors of all outcomes will be blinded to the group allocation of participants, which will not be revealed during the participation of the study.

### Participants

Sixty-six participants diagnosed with severe COPD (FEV_1_ < 50% of predicted; quartiles 3 and 4 in the BODE [Body-mass index, airflow Obstruction, Dyspnoea, and Exercise] index) will be recruited via newspaper advertisements, posters, flyers, visits to local community centres and referral from associated centres. The sample size calculation revealed that based on an α level of 0.05 and a power (1-β) of 0.8, and effect sizes ranging from 0.6 to 1.2 found in similar outcomes (muscle CSA, 6MWT) in our preliminary data after eccentric training, and few studies using combined eccentric and concentric cycling [67–69], a minimum of 17 patients would be necessary per group for this study. However, 22 patients per group will be recruited to account for a typical dropout rate of 30% in long-term training studies. Accredited Ethics Committees from all three Hospitals and universities have approved this study. Any important modification will be informed to the ethics committees. All participants will obtain medical permission from a pulmonary medical specialist before taking part in the study. Exclusion criteria will be: current insulin therapy, long-term oxygen therapy, asthma, other concomitant pulmonary diseases, or other diseases that could affect physical activity [70, 71]. Participants will be instructed to continue with normal everyday activities yet be dis-encouraged from engaging in any unaccustomed training or supplementary nutrition and will be fully informed of the nature and possible risks of all of the experimental procedures before providing their written informed consent. Written informed consent will be obtained from the multicentre coordinator. Criteria for discontinuing intervention will be upon participant request or worsening disease. Patients will receive an initial report for all their clinical assessments, which will be upgraded at the end of the study participation. Participants will be coded as sequential number upon recruitment. A Data Monitoring Committee (DMC) will not be necessary as it will be a short intervention and with sample size that can be handle by the research team. Adverse events will be collected and reported appropriately. All personal information will be coded, and data collected will be kept confidential, and only the PI and multicentre coordinator of this study will have access to the files. The computer will be held in the PI office.

### Characterisation and anthropometric measures of participants

Body mass and height will be determined with a scale and wall-mounted stadiometer (SECA, Germany). The subject´s age, body mass index (BMI), years of diagnosis, smoking habits, comorbidities, and medications will be recorded.

#### Lung function and pulmonary capacity

A spirometry test will be performed to assess the forced vital capacity (FVC) and FEV_1_ from which the FEV_1_/FVC ratio will be derived before and after bronchodilator administration. These measures will be assessed using a digital spirometer (TrueFlow, Switzerland) by a trained certified physiotherapist. The test will be repeated three times, and the best performance will be used for analyses [71].

#### Basal dyspnoea

The magnitude of dyspnoea will be assessed using the Medical Research Council dyspnoea scale; a scale of 5 points where 0 is not dyspnoea and 4 is the perception of patients feeling too breathless to leave the house [72].

#### 6-min walking distance test

The test will be conducted in a level enclosed 20 m corridor. Each participant will be instructed to cover as much ground as possible in six minutes. The test will be performed as recommended [73]. In addition, dyspnoea rates will be assessed at the end of the test.

#### BODE index

The BODE index will be calculated to characterise the patients, and it incorporates BMI, FEV_1_% predicted, score on the modified Medical Research Council dyspnoea scale, and 6-min walk distance to categorise the patients including a more integrated symptomatology method.

### Training protocols

Training will be performed three times per week on each other day, increasing intensity and time progressively over the 12 weeks. Each venue count with automated external defibrillators, oxygen supply, and instructed medical professionals in case of emergency.

*CONC:* The concentric cycling training will consist of 40 min cycling on a calibrated cycle ergometer (Life Fitness, USA). Time will start from 15 min and increase to achieve 40 min in week 4. The workload corresponding to 70% of VO_peak_ achieved during the incremental cycling test will be selected as the target intensity, and it will be monitored and adjusted by the rated perceived exertion (RPE; 6–20 scale), aiming for a target RPE of 14 (“somewhat hard”).

ECC: The eccentric cycling training will consist of 40 min cycling on an eccentric ergometer (Grucox, South Africa). Time will start from 15 min and increase to 40 min in week 4. The intensity will gradually increase to aim for an RPE = 14 “somewhat hard”. As the participant reaches the “somewhat hard” intensity, it will be maintained until the end of the training period, but power output per training session will increase as training progresses (as shown in previous studies) [35, 36].

*CONC* + *ECC:* This group will aim to perform 20 min of concentric cycling as in CONC and 20 min of eccentric cycling as in ECC in this order.

#### Training monitoring

Dyspnoea rates (1–10 scale), heart rate, and power output will be monitored during all training. Arterial oxygen saturation will also be constantly monitored and will be maintained in the 90% range at the lowest following clinical guidelines recommendations.

### Clinical outcomes

#### Blood samples

Resting venous blood samples will be collected following 12-h overnight fast from the antecubital vein will be collected in two EDTA and two serum vacutainer tubes and will be centrifuged for 10 min at 5000 rpm. Glucose and insulin concentrations, lipid profile (triacylglycerols, total cholesterol, high-density lipoprotein cholesterol, low-density lipoprotein cholesterol) and C-reactive protein will be assessed. Furthermore, inflammatory markers (IL-6, IL-1β, and TNF-α) and plasma microRNAs will also be analysed.

#### Functional performance

The timed up-and-go test will be performed to assess functional performance in addition to the 6MWT explained above. In brief, patients will be given verbal instructions to stand up from a chair, walk three meters as quickly and as safely as possible, turn around, walk back, and sit down. The test will be repeated three times, and the best performance will be used for analyses [74].

#### Quality of life and general wellbeing

The Spanish version of the St. George´s Respiratory Questionnaire for COPD patients (SGRQ-C) will be used to measure the participant’s functional health and well-being as in previous studies [75, 76]. It contains 50 items divided into three subscales (i.e., symptoms, activity, and impact), covering a wide range of aspects related with social functioning and psychological disturbances resulting from airways disease.

#### Cross-sectional area (CSA)

Magnetic resonance imaging (MRI) will be performed on both thighs after 15 min lay down rest. MRI images of both thighs will be T1 weighted. Images at 20%, 50%, and 80% distance from the patella and the anterior inferior iliac spine will be used for comparisons of the CSA of different portions of the quadriceps muscle. Whole quadriceps muscle CSA will be measured (ImageJ 1.41, NIH) [67].

#### Maximal voluntary isometric contractions (MVC) strength

Patients will be asked to perform three 3-s MVC of the dominant leg knee extensor muscle at 90° of knee extension; greater force out of three attempts will be used for analyses.

#### Maximal aerobic capacity

The peak oxygen consumption (VO_2peak_) and maximal concentric power output (PO_max_) will be determined during an incremental cycling test to exhaustion using a recumbent cycle ergometer (Livestrong, LS 5.0R model, USA) following the recommendation for COPD [77], using a breath-by-breath metabolic cart (Medisoft, Belgium).

#### Physical activity and sedentary behaviour

Habitual activity level will be assessed using a small, lightweight tri-axial accelerometer (Axivity, Ltd.) worn on the wrist for 24 h/day over seven days to determine the quantity and quality of movement and activity, e.g., activity counts and intensity, step count, sedentary time, number and duration of walking and sitting bouts. This measure will be taken during the first and last week of training [78].

### Molecular analyses

A subgroup of participants will undertake muscle biopsies (n = 30). This sample size was estimated using data from previous studies [79] in which muscle fibre type [26, 28], MRF (i.e., myostatin, MyoD) [26, 32], and phosphorylation of p70S6K [32, 80] increased after training interventions (i.e., aerobic and resistance training). Thus, based on effect sizes ranging from 1 to 2.5 from those studies and an α level of 0.05 and a power (1-β) of 0.8, it was found that eight patients per group would be sufficient. To account for a possible 30% dropout, we will recruit ten patients per group in this study. Muscle biopsies will not be performed on individuals on anti-platelet or anti-coagulation therapy [81].

#### Muscle biopsy

Patients will attend the Laboratory at 8:00 a.m. after overnight fasting without carrying out any type of exercise in the previous 48 h. Muscle samples will be obtained on the vastus lateralis of the non-dominant leg by an experienced medical doctor using the modified Bergstrom needle technique with suction following suggested procedures in a similar population [82]. Muscle samples will be separated from any fatty tissue and blood, and divided into portions that will be treated for specific analyses. Muscle samples (~ 80 mg specimen) will be divided into five parts 1) fibre type isolation (20 mg), 2) microRNAs (20 mg), 3) mRNA expression (10 mg), 4) fibre type morphology (10 mg), and 5) whole muscle protein expression by Western blot (20 mg) analyses. All samples will be stored at − 80 °C until analysis.

### Single muscle fibre isolation

Muscle samples will be freeze-dried in a tissue lyophiliser for 48 h. Next, a small segment (~ 3 µm) will be dissected under a light microscope using forceps and collected into a 10 µl loading buffer (1 × Lammelli’s buffer containing 10% β-mercaptoethanol), and stored overnight at − 80 °C. 30–60 single fibre segments will be collected from each sample as optimised previously [83]. Muscle fibre typing will be assessed by dot blotting. For each subject sample, a minimum of 9 muscle fibre segments will be pooled before electrophoresis.

Whole homogenate muscle specimen and isolated fibre type pools (type I, IIa and IIx) for the canonic protein synthesis Akt/mTOR/S6K1/rpS6 pathway, myogenic regulatory factors (Myogenin, MyoD, myf5, MRF4, and myostatin) and degradation (autophagy; LC3, p62, Parkin, beclin and ubiquitin–proteasome system; MuRF-1, atrogin-1, Need-1/4) systems will be analysed. Furthermore, NF-kB and MAPK pathways will also be explored for a relationship between inflammation and degradation systems. The protein content will be evaluated by Western blotting, and mRNA expression will be assessed by real-time qPCR before and after training.

### Muscle and plasma microRNAs

MicroRNAs will be isolated from plasma samples using the miRNeasy mini kit (Qiagen, CA) and from skeletal muscle tissue samples using the mirVana miRNA Isolation Kit (Life Technologies, USA) according to the manufacturer's instructions. Synthetic spike-in (UniSp3, UniSp4, UniSp5, UniSp6) and synthetic C. elegans miRNA (Cel-miR-39 and Cel-miR-54) will be used to analyse the robustness of the RNA isolation process and quality of isolated microRNA for muscle and plasma samples, respectively. Both extracted RNA will be subjected to Reverse Transcriptase PCR using the TaqMan MicroRNA Reverse Transcription Kit (Applied Biosystems). MicroRNA levels will be measured by real-time PCR amplification using TaqMan MicroRNA Assays specific for miR-1, miR-133, miR206, miR29b, miR-486, miR-499, miR-542, miR-181, miR-133a, miR-27a, miR-181a, miR-208 (Applied Biosystems), according to the manufacturer's instructions. We will also use the red blood cell-specific miR-451 and the stable miR-23a to monitor haemolysis in plasma samples. Furthermore, the results will be normalised to the exogenous spiked in control previously described for each sample.

### Muscle fibre morphology

Muscle sample will be prepared using isopentane snap freezing for immunohistochemical staining for fibre type identification. Overnight incubation will be conducted with the primary antibodies: dystrophin rabbit polyclonal antibody (1:200) (Thermo Scientific, China) and α-II spectrin rabbit polyclonal antibody (1:400), to target the cell membrane and the mouse monoclonal antibody for myosin heavy chain (MyHC, 1:400) to target type II myofibers. After confocal microscopy imaging, fibre type identification and CSA of fibres will be performed automatically using the free available software Fiji-ImageJ (NIH) following previous recommendations [84].

### Statistical analysis

The primary analysis will follow an intent-to-treat approach. A two-way analysis of variance (ANOVA) will be used to compare the three intervention groups and time (Pre- and Post-). If an interaction effect is found, a Tuckey´s post hoc test will be used for pairwise comparisons. Differences will be considered statistically significant at the 0.05 level (two-sided).

## Discussion

This project attempts to elucidate the effects of exercise training on muscle function and many clinical outcomes and a mechanistic approach to understand the muscle mass growth regulation in COPD patients. Although its methodology is straightforward, some debate existed regarding participant blinding and the need for a control group.

COPD is one of the most common chronic diseases worldwide, with an estimated 390 million people affected, with a global prevalence of 10.3% [85]. Although effective medications are available, COPD remains incurable and poorly controlled for numerous patients in many countries, rehabilitation has shown to improve quality of life and reduce mortality. Understanding muscle mass and function loss and recovery after exercise interventions is important to design the best clinical treatment. The effectiveness of clinical interventions in COPD is scarce. Thus, this study will compare three modalities of exercise training (eccentric, concentric and eccentric-concentric groups) in a clinical context in three hospitals in the region of Santiago, Chile. These hospitals will be implemented with the devices to perform eccentric cycling training within the public health system. This will help to support and update the Chilean COPD management guidelines.

### No control and no blinding

Due to ethical considerations, it was decided not to include a control group to provide an effective beneficial intervention (i.e., training) to all participants. We believe that comparing the three exercise interventions with a within- and between- group analysis approach will provide enough information to determine the effectiveness of these interventions for COPD patients on the included outcomes. Furthermore, as eccentric and concentric cycling exercises are different and depictable for patients and therapists, an intervention blinding is not possible with these kinds of interventions. However, assessors and biostatisticians will be blinded to the intervention, which should reduce detection and interpretation bias. The research group will be blinded when interpreting data and writing the conclusions.

### Implication for practice

Muscle cross-sectional area, muscle function, functional performance, aerobic capacity, daily physical activity, and quality of life will be assessed before and after training. Muscle biopsies of vastus lateralis will be performed before and after training in severe COPD patients, from which muscle protein synthesis and degradation pathways will be analysed in a fibre specific manner.

The results from this study will provide important knowledge regarding the treatment of patients with COPD who are unable or non-compliant with conventional offered COPD rehabilitation settings as concentric cycling (i.e., endurance training) is the most provided exercise intervention for these patients. The study will also contribute to the existing knowledge regarding molecular mechanisms involved in the regulation of muscle mass in these patients.

### Ethics and dissemination

Regarding the muscle biopsies, we decided to perform this procedure in a subgroup of each training group to minimise the number of patients undertaking an invasive procedure. We will ensure that all patients receive the best medical attention before, during, and after the biopsy. Before a patient is eligible for muscle biopsy, a complete medical screening will be performed to assess any risks. Furthermore, although it has been reported that complications after muscle biopsies in VL are very low and rare (0.17%, please see Refs. [81, 86]). The most frequently reported complication was local skin infection (0.06%), and in most of these cases, there was a subject error that contributed to the complication (e.g., occlusive dressing, stitch left in too long). The participant will be advised regarding post-biopsy care and given a written copy of the care instructions. The participant will be called the day following the biopsy to assess any related symptoms and generally seen within 1–3 days for the initiation or resumption of exercise training. All biological samples will be kept frozen at − 80 °C for up to 5 years, after which this will be discarded.

Findings of this study are planned to be published in peer-reviewed journals and presented in international conferences with data regarding (1) functional and physiological outcomes, (2) muscle fibber morphology changes after exercise interventions, (3) molecular markers of protein synthesis and degradation changes after interventions in COPD patients, and (4) relationship between miRs from plasma and skeletal muscle samples from COPD patients.

## Data Availability

Not applicable.

## Abbreviations

COPD: Chronic obstructive pulmonary disease
CSA: Cross-sectional area
6MWT: Six-min walking test
TUG: Timed up and go
PR: Pulmonary rehabilitation
ECC: Eccentric cycling training
CONC: Concentric cycling training
ECC + CONC: Eccentric and concentric cycling training
Akt: Protein kinase B
mTOR: Mammalian target of rapamycin
p70s6K: Ribosomal protein S6 kinase beta-1
rS6: Ribosomal Protein S6
MRFs: Myogenic regulatory factors
miRs: MicroRNAs
MyoD: Myoblast determination protein 1
MRF4: Myogenic regulatory factor 4
MuRF-1: Muscle RING-finger protein-1
UP: Ubiquitin–proteasome system
FEV_1_: Forced expiratory volume in 1 s
BODE: Body-mass index, airflow Obstruction, Dyspnoea, and Exercise index
BMI: Body mass index
FVC: Forced vital capacity
VO_peak_: Peak oxygen consumption
PO_max_: Maximal concentric power output
RPE: Rated perceived exertion
EDTA: Ethylenediamine tetraacetic acid
Rpm: Revolutions per minute
IL-6: Interleukin 6
IL-1β: Interleukin 1 beta
TNF-α: Tumour necrosis factor-alpha
SGRQ-C: St. George’s Respiratory Questionnaire for COPD patients
MRI: Magnetic resonance imaging
NIH: National Institute of Health, USA
MVC: Maximal voluntary contraction
mRNA: Messenger ribonucleic acid
LC3: Microtubule-associated protein 1A/1B-light chain 3
p62: Sequestosome 1
NF-kB: Nuclear Factor kappa-light-chain-enhancer of activated B cells
MAPK: Mitogen-activated protein kinase
qPCR: Quantitative polymerase chain reaction
ANOVA: Analysis of variance
VL: Vastus lateralis
